# IL-17A Enhances Retinal Neovascularization

**DOI:** 10.3390/ijms24021747

**Published:** 2023-01-16

**Authors:** Brooklyn E. Taylor, Chieh A. Lee, Thomas E. Zapadka, Amy Y. Zhou, Katherine G. Barber, Zakary R. R. Taylor, Scott J. Howell, Patricia R. Taylor

**Affiliations:** 1 Department of Ophthalmology and Visual Science, Case Western Reserve University School of Medicine, Cleveland, OH 44106, USA; 2 Louis Stokes Cleveland VA Medical Center, Cleveland, OH 44106, USA

**Keywords:** IL-17A, neovascularization, retina

## Abstract

Retinal neovascularization occurs in proliferative diabetic retinopathy, neovascular glaucoma, and age-related macular degeneration. This type of retinal pathology normally occurs in the later stages of these ocular diseases and is a prevalent cause of vision loss. Previously, we determined that Interleukin (IL)-17A plays a pivotal role in the onset and progression of non-proliferative diabetic retinopathy in diabetic mice. Unfortunately, none of our diabetic murine models progress to proliferative diabetic retinopathy. Hence, the role of IL-17A in vascular angiogenesis, neovascularization, and the onset of proliferative diabetic retinopathy was unclear. In the current study, we determined that diabetes-mediated IL-17A enhances vascular endothelial growth factor (VEGF) production in the retina, Muller glia, and retinal endothelial cells. Further, we determined that IL-17A can initiate retinal endothelial cell proliferation and can enhance VEGF-dependent vascular angiogenesis. Finally, by utilizing the oxygen induced retinopathy model, we determined that IL-17A enhances retinal neovascularization. Collectively, the results of this study provide evidence that IL-17A plays a pivotal role in vascular proliferation in the retina. Hence, IL-17A could be a potentially novel therapeutic target for retinal neovascularization, which can cause blindness in multiple ocular diseases.

## 1. Introduction

Diabetic retinopathy is the most common diabetes complication of the microvasculature. Approximately 10,000 diabetics lose their vision due to late-stage diabetic retinopathy annually [1]. Due to hyperglycemic spikes, genetic predispositions, and chronic inflammation, the micro-vasculature of the retina becomes impaired. This can lead to capillary nonperfusion in the retina and the onset of early-stage retinopathy—referred to as non-proliferative diabetic retinopathy [2]. Vascular impairments of the retina such as vascular permeability, capillary nonperfusion, and microaneurysms are the clinical hallmarks of non-proliferative diabetic retinopathy [2,3]. Non-proliferative diabetic retinopathy can progress in stages of mild, moderate, or severe, whereas retinopathy at the mild-moderate stage is primarily asymptomatic [4]. Vision impairment can occur in the severe stage of non-proliferative diabetic retinopathy [4,5]. During early-stage non-proliferative diabetic retinopathy, VEGF production and angiogenic signaling can be induced [2,3]. This can then lead to a more advanced stage of retinopathy known as proliferative diabetic retinopathy. The most prevalent clinical hallmark of proliferative diabetic retinopathy is retinal neovascularization, which can induce severe vision loss [5,6,7].

VEGF induces angioge of new blood vessels in the retina [4]. Hence, intravitreal injections of anti-VEGF are one of the most common treatments for late-stage proliferative diabetic retinopathy [4]. Although anti-VEGF treatments benefit many diabetics with proliferative diabetic retinopathy, there are still ~40% of diabetic retinopathy patients that do not respond to these treatments [4,8]. Hence, there is still a great need for alternative diabetic retinopathy therapeutics. In our previous studies, we provided evidence that IL-17A could be a good therapeutic target for diabetic retinopathy [9,10]. However, all our studies focused on early-stage non-proliferative diabetic retinopathy. Rodent diabetic models are stellar in replicating non-proliferative diabetic retinopathy. However, due to the short life span of rodents, none of these murine models progress into proliferative diabetic retinopathy [11]. In lieu of a proliferative diabetic retinopathy model, proliferation and neovascularization can be examined in chemical or laser-induced neovascularization models [12,13]. In the current study, we examined the role of IL-17A in vascular proliferation and neovascularization using in vitro angiogenic assays and the murine oxygen induced retinopathy (OIR) model. We hypothesized that IL-17A would induce retinal neovascularization directly and indirectly by enhancing VEGF production.

Thus, the objective of this current study was to determine the role of IL-17A in retinal neovascularization. First, the role of IL-17A in VEGF production was investigated in Type I and II diabetic mice. Wherein diabetes-mediated IL-17A production was inhibited by: an anti-IL-17A intravitreal injection in STZ-Type I diabetic mice, weekly intraperitoneal injections of anti-IL-17A in db/db-Type II diabetic mice, or systemically ablated in IL17A^-/-^ STZ-diabetic mice. Additionally, IL-17A-induced VEGF production was examined in murine and human Muller glia and retinal endothelial cells. Next, the role of IL-17A in human retinal endothelial cell proliferation was determined using ex vivo angiogenesis assays and BrdU analysis. Finally, the role of IL-17A in retinal neovascularization was examined in IL17A^-/-^ and C57BL/6 controls using the OIR model. Collectively, the findings define the role of IL-17A in retinal neovascularization.

## 2. Results

### 2.1. Hyperglycemia in STZ-Induced and Lepr^db^ Diabetic Mice

Hyperglycemic conditions were confirmed 17 days after the last streptozotocin (STZ) injection by 6 h fasted blood glucose levels in the STZ-induced diabetic mice. Alternatively, hyperglycemic conditions were confirmed in Lepr^db^ (db/db) mice at 6 weeks of age via 6 h fasted blood glucose levels. All mice had a blood glucose level at or above 500 mg/dL. To confirm that all mice were hyperglycemic throughout the timeline of the study, glycated hemoglobin A1c was measured 6 and 22 weeks after diabetes was confirmed. As shown in Table 1, all diabetic mice had significantly higher A1c levels than the non-diabetic controls, and all diabetic mice A1c scores are in the diabetic parameters.

### 2.2. Diabetes-Mediated IL-17A Enhances VEGF Production in the Retina

**Figure 1:**
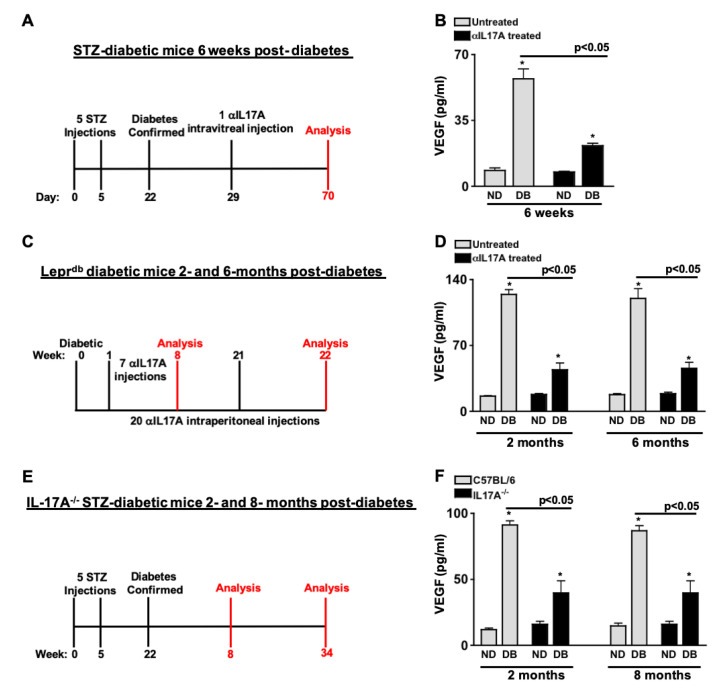
Inhibition of Diabetes-Mediated IL-17A Decreases VEGF Production. (**A**) Schematic of the 6-week analysis of STZ-diabetic C57BL/6 mice (n = 9/group) receiving an anti-IL-17A intravitreal injection. (**B**) Quantification of VEGF in retinas of untreated (grey) and anti-IL-17A treated (black) non-diabetic (ND) and diabetic (DB) mice 6 weeks post-diabetes. (**C**) Schematic of the 2- and 6-month analyses of Lepr^db^ mice (n = 9/group) receiving weekly intraperitoneal injections of anti-IL-17A. (**D**) Levels of VEGF in the retinas of untreated (grey) and anti-IL-17A treated (black) non-diabetic (ND) and diabetic (DB) Lepr^db^ mice 2- and 6-months post-diabetes. (**E**) Schematic of the 2- and 8-month analyses of STZ-diabetic C57BL/6 and IL17A^-/-^ mice (n = 9/group). (**F**) Quantification of VEGF in the retinas of C57BL/6 (grey) or IL17A^-/-^ (black) non-diabetic (ND) and diabetic (DB) mice 2- and 8-months post-diabetes. Error bars represent the SEM; * *p* < 0.01. The *p*-value was first equated by two-way ANOVA analysis and then unpaired Student’s *t*-test with Tukey’s post-hoc analysis.

VEGF plays a pivotal role in the onset of neovascularization in proliferative diabetic retinopathy [14]. To determine if diabetes-mediated IL-17A impacts retinal VEGF production, IL-17A was inhibited in murine models of diabetic retinopathy, and levels of VEGF in retinal protein lysates were quantified by ELISA. As shown in Figure 1A, IL-17A was neutralized in the retina of STZ-diabetic C57BL/6 mice with one 50 μg/mL intravitreal injection of anti-IL-17A; 1 week after diabetes was confirmed. Levels of VEGF were quantified six weeks after the injection. There was a significant increase of VEGF in the retinas of untreated diabetic (DB) compared to non-diabetic (ND) mice. However, levels of VEGF were significantly decreased in the retinas of diabetic mice that received an anti-IL-17A injection (black) compared to untreated diabetic (grey) mice (Figure 1B). Next, weekly intraperitoneal injections of 50 μg/mL of anti-IL-17A were administered to Lepr^db^ (db/db) diabetic mice (Figure 1C). To determine if IL-17A impacts retinal VEGF production in a murine model of Type II diabetes complications, VEGF was quantified in retinal protein lysates 2- and 6-months after diabetic conditions were confirmed. As shown in Figure 1D, the level of VEGF was significantly increased to ~140 pg/mL in the retinas of homozygous db/db diabetic mice (DB) compared to heterozygous non-diabetic controls (ND). Conversely, when IL-17A was systemically neutralized in the db/db diabetic mice (black), VEGF was significantly decreased 2- and 6-months after diabetes was confirmed (Figure 1D). Finally, the retinas of STZ-induced diabetic C57BL/6 (wild-type) and IL17A^-/-^ mice were examined 2- and 8-months after diabetic conditions were confirmed (as displayed in Figure 1E). VEGF is significantly increased in the retina of all diabetic (DB) mice compared to non-diabetic (ND) mice (Figure 1F). Yet, when IL-17A was systemically ablated in the IL17A^-/-^ STZ-diabetic mice (black), VEGF was significantly decreased to ~45 pg/mL 2- and 8-months post-diabetes. This is more than two-fold lower than the ~100 pg/mL of VEGF detected in the retinas of the wild-type STZ-diabetic mice (grey). Overall, these data provide evidence that diabetes-mediated IL-17A enhances VEGF production in the retina.

### 2.3. IL-17A Induces VEGF Production in Retina Cells

**Figure 2:**
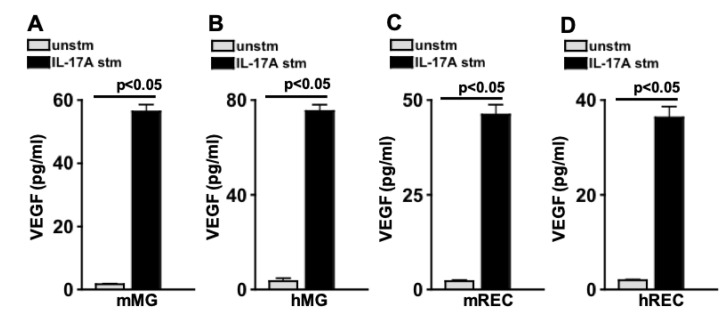
VEGF Production by Retina Cells Stimulated with IL-17A. ELISA quantification of VEGF in spent media of unstimulated (grey) or IL-17A (black) stimulated: (**A**) murine Muller glia (mMG), (**B**) human Muller glia (hMG), (**C**) murine retinal endothelial cells (mREC), and (**D**) human retinal endothelial cells (hREC). Error bars represent the SEM. The *p*-values were equated by two-way ANOVA and then unpaired Student’s *t*-test with Tukey’s post-hoc analysis.

Previously, we reported that Muller glia and retinal endothelial cells express the IL-17A receptor [6]. To determine if IL-17A can induce these retina cells to produce VEGF, cells were stimulated with 20 ng/mL of rIL-17A for 16 h, and levels of VEGF in spent media were quantified by ELISA. As shown in Figure 2, only negligible levels of VEGF were detected in all of the unstimulated (unstm-grey) cells. Mouse Muller glia (mMG) produced ~57 pg/mL (Figure 2A), human Muller glia (hMG) produced ~78 pg/mL (Figure 2B), mouse retina endothelial cells (mREC) produced ~48 pg/mL (Figure 2C), and human retina endothelial cells (hREC) produced ~37 pg/mL (Figure 2D) of VEGF when they were stimulated with recombinant (r)IL-17A (IL-17A stm-black). Taken together, IL-17A can induce these retina cells to produce VEGF.

### 2.4. IL-17A Initiates Retina Endothelial Cell Angiogenesis

**Figure 3:**
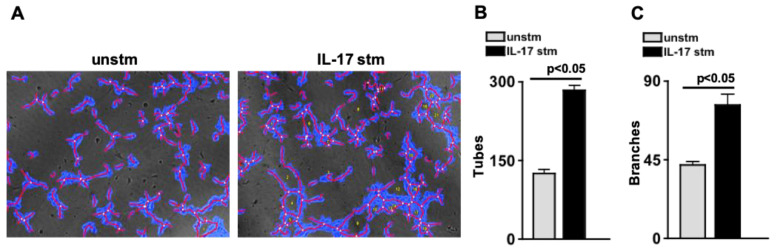
IL-17A Initiates Retinal Endothelial Cell Proliferation. (**A**) Representative images of WimTube analytical overlay of unstimulated and IL-17A-stimulated human retinal endothelial cells. Quantification of the number of angiogenic tubes (**B**) and branches (**C**) in unstimulated (grey) or IL-17A-stimulated (black) human retinal endothelial cells. Error bars represent the SEM. The *p*-values were equated using two-way ANOVA and then unpaired Student’s *t*-test with Tukey’s post-hoc analysis.

To determine if IL-17A can induce vascular angiogenesis, human retina endothelial cells (hREC) were plated on Geltrex, stimulated with 20 ng/mL of rIL-17A for 16 h, and then analyzed by microscopy and WimTube software. As shown in Figure 3A, the covered area (blue), tubes (red), branch points (white dots), and loops (yellow numbers) were analyzed in unstimulated (unstm) and IL-17A-stimulated (IL-17 stm) hREC. All angiogenic parameters were increased in the IL-17A-stimulated versus unstimulated cells. Notably, the number of tubes (Figure 3B) and branch points (Figure 3C) were significantly increased in the IL-17A-stimulated cells. These results provide evidence that IL-17A is sufficient to initiate cellular angiogenesis of retinal endothelial cells.

### 2.5. IL-17A Enhances Retina Endothelial Cell Proliferation

**Figure 4:**
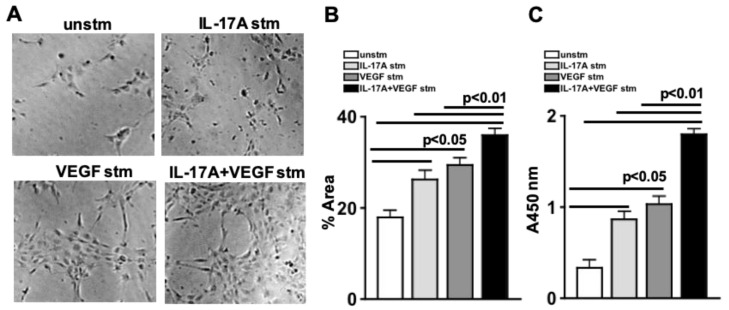
IL-17A Enhances Retinal Endothelial Cell Angiogenesis. (**A**) Representative phase contrast images of unstimulated or stimulated human retinal endothelial cells. Quantification of the percentage of proliferation area (**B**), or BrdU quantification of proliferation (**C**) in unstimulated (white), IL-17A-stimulated (light grey), VEGF-stimulated (dark grey), or IL-17A+VEGF-stimulated (black) human retinal endothelial cells. Error bars represent the SEM. All *p*-values were first equated by two-way ANOVA and then unpaired Student’s *t*-test with Tukey’s post-hoc analysis.

To examine the impact of IL-17A in human retina endothelial cell proliferation, cells were stimulated with 20 ng/mL of rIL-17A, 20 ng/mL of rVEGFA, or 20 ng/mL of rIL-17A+20 ng/mL of rVEGFA. After 16 h of incubation at 37 °C with 5% CO_2_, proliferation was either examined by BrdU or by microscopy and WimTube analysis. As shown, in Figure 4A all stimulated cells (IL-17A stm, VEGF stm, IL-17A+VEGF stm) proliferated in comparison to the non-proliferating unstimulated (unstm) cells. Additionally, the area that the proliferating tubular structure covers (Figure 4B) was significantly increased when the hREC were stimulated with rIL-17A (light grey), rVEGFA (dark grey), or rIL-17A+rVEGFA (black).

In a colorimetric assay, cellular proliferation was detected using a 5-bromo-2′-deoxyuridine (BrdU) assay. BrdU incorporates into the cellular DNA during proliferation and can be detected by a plate reader at an absorbance of 450 nm. When hREC were stimulated with rIL-17A (light grey) or rVEGFA (dark grey), cellular proliferation was significantly enhanced. Further, hREC cellular proliferation was significantly increased in an additive manner when hREC were stimulated with both rIL-17A and rVEGFA (Figure 4C). Collectively, these data provide evidence that IL-17A can both initiate and enhance the VEGF-dependent proliferation of vascular retina cells.

### 2.6. IL-17A Ablation Significantly Decreases Neovascularization in Oxygen Induced Retinopathy

**Figure 5:**
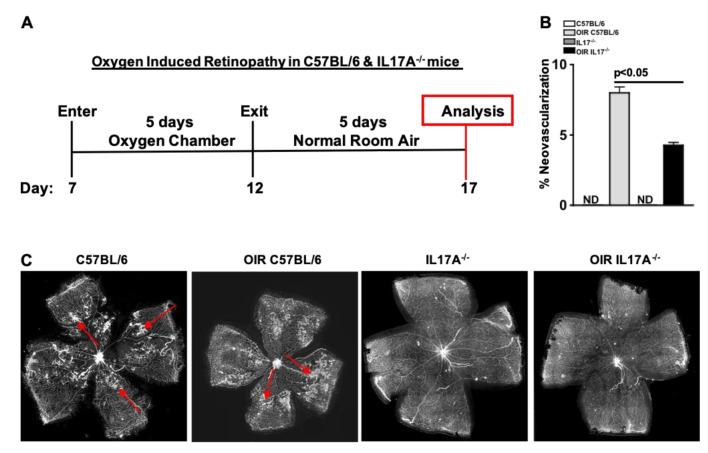
Retinal Neovascularization is Decreased in IL17A^-/-^ Mice with Oxygen-Induced Retinopathy. (**A**) Schematic of the murine model of oxygen induced retinopathy and retinal neovascularization. (**B**) Quantification of neovascularization in C57BL/6 and IL17A^-/-^ mice (n = 5/group). (**C**) Representative images of neovascularization in retina whole mounts. Red arrows highlight neovascularization. ND = not detected. Error bars represent the SEM. The *p*-value was first equated by two-way ANOVA analysis and then unpaired Student’s *t*-test with Tukey’s post-hoc analysis.

The oxygen induced retinopathy model was used to examine the role of IL-17A in retinal neovascularization. Seven-day-old C57BL/6 or IL17A^-/-^ mice (with nursing mother; n = 5 pups/group) were placed into a 75% oxygen chamber for 5 days, then placed into normal room air for 5 more days prior to analysis on day 17 (Figure 5A). Neovascularization was then quantitated in isolectin-stained retina whole mounts, as shown in Figure 5C (red arrows highlight neovascularization).

There was no neovascularization detected (ND = not detected) in the C57BL/6 or IL17A^-/-^ mice that were not placed into the oxygen chamber. However, ~8% of the retina was neovascularized in the C57BL/6 mice that were placed in the oxygen chamber (light grey). Neovascularization was significantly decreased to ~4% of the retina when IL-17A was systemically ablated in the hyperoxia-engaged IL17A^-/-^ (black) mice (Figure 5B). Altogether, this provides evidence that IL-17A plays a pivotal role in retinal neovascularization.

## 3. Discussion

Overall, the data from this study provides evidence that IL-17A plays a pivotal role in retinal neovascularization. Previously, we determined that diabetes-mediated IL-17A enhanced retinal inflammation, vascular leakage, and capillary degeneration [9]. Hence, we identified IL-17A as a potential therapeutic target for early-stage non-proliferative diabetic retinopathy. Yet, none of our diabetes murine models progress to the proliferative stage of diabetic retinopathy. So, it was unclear if IL-17A impacted vascular proliferation of the retina or retinal neovascularization. In other diseases, IL-17A has been shown to enhance VEGF production [15]. Thus, we hypothesized that IL-17A would play an indirect role in retinal proliferation, due to enhanced levels of VEGF. Through in vivo neutralization of IL-17A in murine models of Type I and II diabetes, we did determine that diabetes-mediated IL-17A does enhance VEGF. Thus, IL-17A can enhance retinal proliferation indirectly by enhancing VEGF, which is known to induce vascular proliferation and neovascularization in the retina [14].

Notably, IL-17A was sufficient to induce retinal endothelial cell proliferation and retinal neovascularization. Tubular angiogenesis was induced when human retinal endothelial cells were stimulated with rIL-17A. Further, retinal endothelial cell angiogenesis was enhanced when rIL-17A was added to VEGF-stimulated cells. Not only does this identify a direct role for IL-17A in retinal endothelial cell proliferation, but it also provides evidence that IL-17A impacts VEGF-driven angiogenesis.

In cancer studies, IL-17A has also been shown to play a role in VEGF-driven angiogenesis [15]. Further, it was determined that anti-IL-17A could enhance the efficacy of anti-VEGF treatments in cancer cells that did not respond to anti-VEGF treatments [15,16,17]. So, it is feasible that anti-IL-17A could enhance the efficacy of anti-VEGF treatments for diabetic retinopathy. Many more pre-clinical studies are required to solidify if anti-IL-17A could supplement anti-VEGF treatments for diabetic retinopathy. Future studies will focus on laser-induced neovascularization in IL-17A adoptive transfer murine models, so that conditional IL-17A knock-out and IL-17A knock-in modifications can be examined. Still, our current data provides evidence that anti-IL-17A could be a potentially novel therapeutic for diabetic retinopathy.

To examine retinal neovascularization, wild-type C57BL/6 and IL17A^-/-^ neonatal mice were placed in hyperoxia conditions and then in normal air. These hyperoxia conditions initiate angiogenesis. Further, due to the plasticity of the neonatal eye, blood vessels re-vascularize when placed in normal air conditions [9]. Diabetes-mediated components such as hyperglycemic-driven pathologies cannot be examined in this model. However, the role of IL-17A in neovascularization was defined by utilizing this model. Thus, we cannot fully conclude that IL-17A plays a role in proliferative diabetic retinopathy. Yet, our study does provide evidence that IL-17A can initiate and enhance vascular angiogenesis and impacts retinal neovascularization. These novel findings actually yield a much more clinically relevant result. Because neovascularization enhances retinal pathogenesis in multiple retinal diseases. Such as, age-related macular degeneration and neovascular glaucoma, as well as proliferative diabetic retinopathy [18,19,20]. Hence, the findings of this study provide evidence that IL-17A plays a pivotal role in retinal neovascularization. Further suggesting that IL-17A could be a potentially novel therapeutic target for neovascularization in multiple ocular diseases.

## 4. Materials and Methods

### 4.1. Streptozotocin (STZ)-Induced Diabetic Mice

C57BL/6 (n = 9) and IL17A^-/-^ (n = 9) mice (on C57BL/6 background) were purchased from Jackson laboratories. Diabetes was induced by intraperitoneal injections of streptozotocin (60 mg/Kg-body weight) for 5 consecutive days. Diabetic conditions (hyperglycemia) were confirmed by a 6 h fasted blood glucose (FBG) analysis. Mice were classified as diabetic if they had an FBG >275 mg/dL. Hyperglycemia was further measured using the Crystal Chem Mouse A1c kit (Elk Grove Village, IL, USA). Mice received insulin (0–0.2U) as needed, due to excessive weight loss. Analyses were performed 2- and 8-months after diabetic conditions were confirmed. These time points were chosen because retinal inflammation begins at the 2-month time point, and early-stage diabetic retinopathy begins at the 8-month time point in this diabetes murine model [2,9,10].

### 4.2. Intravitreal Injection of Anti-IL-17A in STZ-Induced Diabetic Mice

Mice that received an intravitreal injection of anti-IL-17A (n = 9 untreated and n = 9 treated mice) were anesthetized and the eye was numbed by proparacaine. The eye was then dilated with tropicamide prior to a beveled 34-gauge needle being inserted ~2 mm posterior to the limbus to directly reach the vitreous cavity. The needle was then replaced with a blunt-tip 34-gauge needle that dispensed 1 μL of saline containing 50 μg/mL of anti-IL-17A neutralizing antibody. Ophthalmic bacitracin-neomycin-polymyxin ointment was applied to the eye daily for 3 consecutive days post-injection. Mice received 1 intravitreal injection of IL-17A, 1 week after diabetes was confirmed. IL-17A was neutralized for 6 weeks. So, analyses were performed 6 weeks after injection.

### 4.3. Lepr^db^ Diabetic Mice

Lepr^db^ homozygous (db/db) and heterozygous controls were purchased from Jackson Laboratory. At ~6 weeks old the homozygous (n = 18) mice spontaneously developed diabetes and became obese (the heterozygous controls (n = 18) have slight weight gain, but not diabetes). Fasted blood glucose levels were used to confirm diabetes. Anti-mouse IL-17A neutralizing monoclonal antibody was injected in the intraperitoneal cavity one time weekly at a concentration of 50 μg/mL. Injections started 1 week after diabetes was confirmed. At the 2-month analysis time point, mice received 7 injections. At the 6-month analysis time point, mice received 20 injections. Analyses were made at these time points because retinal inflammation begins at the 2-month time point, and early-stage diabetic retinopathy begins at the 6-month time point in this murine diabetes model [21].

### 4.4. VEGF ELISA Analysis

Levels of VEGF in cellular spent media or protein lysates of the retinas (n = 9/group) from C57BL/6, IL17A^-/-^, or db/db mice were analyzed by a VEGF sandwich ELISA according to the manufacturer’s directions (R&D Biosciences, Minneapolis, MN, USA).

### 4.5. Human Muller Glia

Muller glia were isolated from the posterior section of the retinal globe of human cadaver eyes (Eversight, Cleveland, OH, USA). Retinas were mechanically disrupted and incubated in DMEM/HAM F12 media at 37 °C with 5% CO_2_ for at least 2 passages of cells. Cells were analyzed by flow cytometry to confirm that the Muller glia were >95% GLAST^+^/Vimentin^+^ pure.

### 4.6. Murine Muller Glia

Primary Muller glia were isolated from the retinas of 3-week-old C57BL/6 mice, as previously described [22]. Eyes were removed, enucleated, and the posterior cup was isolated. Retinal pigment epithelial cells were removed, the posterior cup was digested in papain, and incubated at 37 °C with 5% CO_2_ for 7 days. After passage 3, adherent Muller glia purity was confirmed by flow cytometry analysis (cells were >95% GLAST^+^/Vimentin^+^ Muller glia).

### 4.7. Human Retinal Endothelial Cells

Primary human retinal endothelial cells of the retinal microvasculature were purchased from Cell Systems. Cells were maintained in manufacturer’s media as instructed.

### 4.8. Murine Retinal Endothelial Cells

These cells were a kind gift of Nader Sheibani. The Sheibani lab isolated the endothelial cells as previously described [23,24]. Briefly, murine retina endothelial cells were isolated from the retinas of C57BL/6 mice. Retinas were digested in collagenase; isolated retina cells were then CD144^+-^ sorted via positive selection by magnetic beads. Cell purity of >99% VE-Cadherin+ retinal endothelial cells was confirmed by flow cytometry analysis.

### 4.9. Angiogenesis Assay

In vitro tube formation was performed as previously described [25]. Briefly, human retinal endothelial cells were cultured in complete media until ~95% confluent. Assay plates were prepared with a thin layer of reduced growth factor basement membrane matrix (Gibco, Detroit, MI, USA). Cells were serum starved for one hour prior to passage and then plated in minimal media. IL-17A (20 ng/mL) and/or VEGFA (10 ng/mL) was added to wells 30 min after plating. Plates were incubated overnight. Phase contrast images were taken at 5× zoom and angiogenesis was quantified using Wimtube software (Wimasis, Cordoba, Spain).

### 4.10. BrdU Proliferation Assay

Cell proliferation was measured using the BrdU Cell Proliferation Kit (Cell Signaling Technology, Danvers, MA, USA), per the manufacturer’s instructions. Human retinal endothelial cells were serum starved for one hour prior to passage, and then plated at 10,000 cells per well in a 96-well plate with 1× BrdU. Plates were incubated at 37 °C with 5% CO_2_ for 16 h, and readout was obtained at Absorbance-A450 on the plate reader immediately after development.

### 4.11. Oxygen Induced Retinopathy

Postnatal Day (P)7 (date-of-birth = P0) neonatal mice (n = 5/group) along with their mother are placed in a 75% oxygen chamber (BioSpherix ProOx Model 110, Parish, BioSpherix Ltd, NY, USA) and held for 5 consecutive days in hyperoxia. P12 mice are then returned to normal room air and held for 5 days. At P17, neonatal mice are weighed (underdeveloped mice are excluded), euthanized via anesthetic overdose, and eyes enucleated. Eyes are transferred to 4% PFA to fix for 1 h. Fixed eyes are washed three times in PBS, then the retinas are isolated, wet mounts are made, and stained with isolectin. Images of isolectin-stained, whole-mount retinas were collected using a 20× objective on a Zeiss Axio Scan Z1. Retinal whole-mount images were then imported into Metamorph Imaging software for analysis. Briefly, the entire area of the retina was determined via tracing. Next, the area of neovascularization was determined by using a combination of thresholding, size discrimination, and manual removal based on the isolectin staining. The resulting neovascularization value was divided by the area of the retina to produce the percentage of neovascularization shown in Figure 5B. Representative images were converted to black and white, for better visibility of neovascularization in Figure 5C. Each retina was independently measured by three graders and the resulting data was averaged.

### 4.12. Statistical Analysis

Statistical analysis was performed using a two-way ANOVA analysis and an unpaired Student’s *t*-test with Tukey’s post-hoc analysis (Prism Version 9, GraphPad Software, San Diego, CA, USA). A *p*-value < 0.05 was considered significant.

## 5. Conclusions

In conclusion, diabetes-mediated VEGF production was significantly decreased when IL-17A was ablated or neutralized in murine models of Type I and II diabetes. Additionally, Muller glia and retinal endothelial cells produced VEGF when they were stimulated with recombinant IL-17A. Further, human retinal endothelial cells proliferated when stimulated with rIL-17A. Finally, by using the OIR model, we determined that neovascularization was significantly decreased in the retinas of IL17A^-/-^ mice when compared to C57BL/6 controls. Hence, these results provide evidence that IL-17A plays a pivotal role in retinal neovascularization.

## Figures and Tables

**Table 1:** Clinical data of non-diabetic and diabetic mice.

Group	%HbA_1c_ (Week 6)	%HbA_1c_ (Week 22)
**STZ-diabetic mice 6 weeks after αIL17A intravitreal injection**		
αIL17A C57BL/6 ND	5.3 + 0.3	N/A
αIL17A C57BL/6 DB	12.0 + 1.9 *	N/A
C57BL/6 ND	5.4 + 0.1	N/A
C57BL/6 DB	11.8 + 1.9 *	N/A
**Wild-type and IL-17A^-/-^ STZ-diabetic mice 2- and 8-months post-diabetes**		
IL17^-/-^ ND	4.9 + 0.3	5.3 + 0.3
IL17^-/-^ DB	11.2 + 2.1 *	12.0 + 1.5 *
C57BL/6 ND	4.8 + 0.6	5.0 + 0.3
C57BL/6 DB	12.2 + 0.8 *	12.6 + 0.8 *
**αIL17A injected db/db diabetic mice 2- and 6- months post-diabetes**		
αIL17A Lepr^db^ Heterozygous ND	4.9 + 0.6	5.0 + 0.3
αIL17A Lepr^db^ Homozygous DB	12.8 + 2.1 *	13.3 + 1.5 *
Lepr^db^ Heterozygous ND	5.1 + 0.2	4.8 + 0.3
Lepr^db^ Homozygous DB	12.7 + 1.1 *	12.8 + 1.0 *

Data are mean ± SD; n = 9 mice/group. ND = non-diabetic, DB = diabetic, and N/A = not applicable (22 weeks is past the time of harvest for that study). * *p* < 0.01, wherein the *p*-value was first equated by two-way ANOVA and then unpaired Student’s *t*-test with Tukey’s post-hoc analysis.

## Data Availability

The dataset generated during and/or analyzed during the current study is available from the corresponding author upon reasonable request.
